# Effects of 1 Year of Lifestyle Intervention on Institutionalized Older Adults

**DOI:** 10.3390/ijerph18147612

**Published:** 2021-07-17

**Authors:** Daniele Magistro, Fabio Carlevaro, Francesca Magno, Martina Simon, Nicola Camp, Noel Kinrade, Massimiliano Zecca, Giovanni Musella

**Affiliations:** 1 Department of Sport Science, School of Science and Technology, Nottingham Trent University, Nottingham NG11 8NS, UK; nicola.camp@ntu.ac.uk (N.C.); noel.kinrade@ntu.ac.uk (N.K.); 2 Polo Universitario Asti Studi Superiori (Uni-Astiss), 14100 Asti, Italy; fabio.carlevaro@unito.it (F.C.); francesca.magno@unito.it (F.M.); martina.simon@unito.it (M.S.); giovanni.musella@unito.it (G.M.); 3 Dipartimento di Scienze della Vita e Biologia dei Sistemi, University of Torino, 10124 Torino, Italy; 4 Wolfson School of Mechanical, Electrical and Manufacturing Engineering, Loughborough University, Loughborough LE11 3TU, UK; m.zecca@lboro.ac.uk

**Keywords:** physical activity, mobility, physical functioning, ADL, depression, dementia

## Abstract

The socio-economic and health consequences of our ageing population are well documented, with older adults living in long-term care facilities amongst the frailest possessing specific and significant healthcare and social care needs. These needs may be exacerbated through the sedentary behaviour which is prevalent within care home settings. Reducing sedentary time can reduce the risk of many diseases and improve functional health, implying that improvements in health may be gained by simply helping older adults substitute time spent sitting with time spent standing or in light-intensity ambulation. This study identified the impact of 1 year of lifestyle intervention in a group of older adults living in a long-term care setting in Italy. One hundred and eleven older adults (mean age, 82.37 years; SD = 10.55 years) participated in the study. Sixty-nine older adults were in the intervention group (35 without severe cognitive decline and 34 with dementia) and 42 older adults were in the control group. Data on physical functioning, basic activities of daily living (BADL) and mood were collected 4 times, before, during (every four months) and after the 1 year of intervention. The lifestyle intervention focused on improving the amount of time spent every week in active behaviour and physical activity (minimum 150 min of weekly activities). All participants completed the training program and no adverse events, related to the program, occurred. The intervention group showed steady and significant improvements in physical functioning and a stable situation in BADL and mood following the intervention in older adults with and without dementia, whilst the control group exhibited a significant decline over time. These results suggest that engagement in a physical activity intervention may benefit care home residents with and without dementia both physically and mentally, leading to improved social care and a reduced burden on healthcare services.

## 1. Introduction

Older age is associated with many socio-economic and health consequences, including an increased risk of many diseases, poor physical function and frailty, cognitive decline and other forms of disability [1,2]. Older adults living in long-term care facilities are amongst the frailest in our population, with specific and significant healthcare and social care needs [3]. The number of older adults who are unable to live independently due to healthcare needs is increasing rapidly, which poses serious difficulties for health services and social policies due to the high costs associated with their daily care [4]. Indeed, in Europe, one of the EU’s key priorities is to deliver better health and social care outcomes, whereby having high quality and fit-for-purpose care homes is fundamental for providing high quality, safe and efficient social care [5]. Within this, there are two key aspects: maintenance of an active lifestyle, due to the positive effect on the well-being of the residents [6]; and maintenance of resident autonomy, which is a universal right that must be guaranteed by care facilities [7]. Therefore, nursing and care homes must identify the best strategies to promote an active lifestyle of their residents whilst also maintaining their autonomy [5].

In general, older adults are the most sedentary group within society, with two thirds of their awake time per day spent in sedentary activities [8,9]. This could be exacerbated in non-independent older adults that are living in a care facility. It is reported that care-home residents spend the majority of their time inactive, with low levels of interaction with staff [10,11]. Where, considering personal status and the environment, physical activities are limited and the sedentary lifestyle became predominant, the adverse consequences of low activity levels such as depression and poor mobility are prevalent in older care-home residents [11], with most residents experiencing excessive sedentary lifestyle [3]. Whilst research has yet to directly measure physical activity of older adults in care facilities, it is plausible to assume that this would fall below, or barely above the ‘threshold’ of minimum physical activity due to several limiting factors. These include greater prevalence of fear of falls, joint pain, decreased physical function and other contraindications that may limit motivation and ability to engage in many purposeful activities. Moreover, for older adults living in care institutions, abilities and resources for initiating or maintaining health behaviours are more restricted than their community-dwelling counterparts. They are very likely to encounter environmental barriers to physical activity, so they are at higher risk of being physically inactive [12]. Moreover, because of the frail characteristics of older residents and the dependency-producing environments of care institution settings, institutionalised older adults have often not been viewed as a prime target for health promoting programmes [13,14].

Previous research suggests that reducing sedentary time can reduce the risk of many diseases and improve functional health [15,16]. This suggests that potential improvements in health may be gained by simply helping older adults substitute time spent sitting with time spent standing or in light-intensity ambulation. Indeed, recent evidence has shown that many of deleterious factors associated with older age are ameliorated by an active life and higher levels of physical activity [17,18,19,20,21,22]. Moreover, engaging in a physical activity and having an active life is one of the first steps to reduce functional decline and fear of falling [23], especially in particularly vulnerable individuals, such as people living in long-term care settings. Previous literature [24,25,26,27,28,29] suggests that physical training programs, such as balance and functional training can improve balance and mobility, Activity of Daily Living (ADL) performance and the general quality of life in older adults living in long-term care settings.

The purpose of this study was to determine the effect of 1 year of lifestyle intervention on physical functioning, basic ADL and mood in older adults living in a long-term care setting. The intervention was focused on improving the amount of time spent being physically active weekly.

## 2. Materials and Methods

### 2.1. Design of the Study

**Figure 1:**
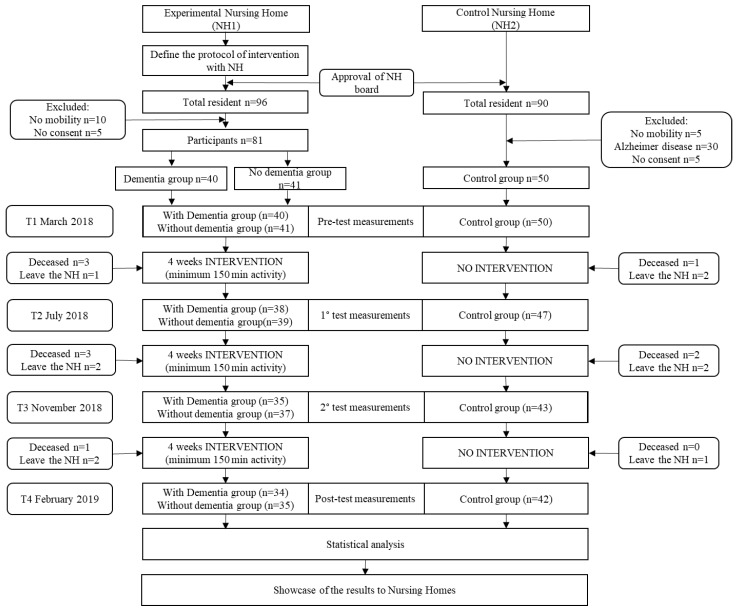
Recruitment process and attendance information of the study population.

The present study was a non-randomised, quasi-experimental longitudinal intervention study with one intervention group and a control group, with participants recruited from two nursing homes. The intervention took place in a Nursing Home (NH1), in the north of Italy. All the residents of the facility were involved in the activity, forming the intervention group with older adults with and without dementia. The older adults of the control group were recruited in a different Nursing Home (NH2), with the same features of the aforementioned. Both experimental groups carried out the same physical activity program for a year, while the control group continued with their usual routine. During the experimental year, control tests were carried out every four months for a total of four tests: a pre-test before the starting, a post-test at the end of the intervention and two mid-tests, one in the fourth and one in the eighth month. All the measures were undertaken in both nursing homes during the same period. Figure 1 shows the study design. The physical functioning tests were implemented by a physical activity professional, while the questionnaires were implemented by a professional psychologist. The ethical committee of the University of Torino approved the study. Participants were informed that their participation in the study was voluntary and confidential. Participants gave their informed written consent to participate in the study in accordance with the local medical ethics committee and in compliance with the ethical standards provided in the 1964 Declaration of Helsinki.

### 2.2. Participants

All 96 older adults living in NH1 were involved in this study. The inclusion parameters were restricted to the possibility of participating in the proposed activity and to the will of subscribing to the project. A total of 10 people were excluded from the intervention due to the severity of their condition and being unable to walk, 5 people did not agree to participate in the activity, while the other 81 were admitted to the experimental phase. The experimental groups consisted of 40 older adults with dementia and 41 older adults without cognitive disease. The control group were recruited from a similar facility (NH2); 50 older adults were recruited for this group. Due to the nature of longitudinal study and the age of the studied population the rate of dropout attrition from pre-test and post-test was around 15% (see flow diagram of the study, Figure 1). All the socio-demographic features of the three groups are shown in Table 1.

### 2.3. Intervention

The intervention was designed in agreement with the WHO physical activity recommendations for older adults [30,31,32] and in accordance with the recommendations on physical activity for older adults living in long-term care facilities [33], suggesting that they also relate to older adults affected by dementia. Therefore, the activities were designed so that a minimum of 150 min of physical activity was reached every week for each participant in the study. The minimum of 150 min of weekly activity were divided into three main types: group physical activity lessons (two times per week), with a physical education professional with exercises focused on muscular strength, gait and balance; individual activities with a physiotherapist for strength maintenance and articular mobility (one time per week); activities with a professional educator for older adults and health staff, focused on educational and recreational activities (two times per week). All the activities were engaged in for 5 days a week to ensure a minimum of 30 min a day of activity for each participant. However, the duration of the activities variated each time based on the daily plan of the NH and the availability of the staff. The instructor together with the NH1 staff monitored adherence to the overall activity. The intensity of the different physical activities was checked by using the exertion rating scale and the talk test during the activities.

### 2.4. Measurements

Tinetti Test [34,35] was used to assess mobility, balance, gait and fall risk. It is a performance-oriented test divided into two parts: a balance specific section scored on a range of 0 to 16 points and a gait specific section scored on a range of 0 to 12 points for a total score between 0 and 28 points, where lower scores indicate a problem in balance and gait and higher risk of falling.

Borg CR10 scale [36,37] was used to assess exertion through measuring the self-perceived fatigue after a physical exercise. In this case the researcher asked the participants to indicate their level of exertion after the Tinetti Test. The score ranged from 0 to 10, with 0 indicating “no exertion at all” and 10 indicating “maximal exertion”.

Arm curl test [38,39]: this was used to measure upper extremity muscle strength. The participant, while in a sitting position, starts with a full elbow extension of the right arm and lifts the weight, flexing the elbow to full flexion, then it repeats the test with the left arm. In the test the weight lifted was 8 pounds (3.63 Kg) for men and 5 pounds (2.27 Kg) for women. The score was the number of lifts performed in 30 s for each arm.

Back scratch test [40]: the test was used to measure the flexibility and the mobility of the shoulders and the upper extremities. The participant is asked to stand up and place the non-preferred arm flexed behind the back, below the shoulder, with the elbow to full flexion and the palm of the hand facing outwards, the preferred arm over the same shoulder with the elbow to full flexion and the palm of the hand facing the back. The examiner asks the participant to overlap the preferred hand with the second one, the distance between the two middle fingers is measured with a tape. If there was distance between the middle fingers, the score would be negative; if the fingers touched each other the score would be zero and if the fingers were overlapping the score would be positive. The test was repeated switching the position of the arms.

Hand grip teats [41] was assessed with Baseline© Smedley digital hand dynamometer (in kilogram (kg). The participant was asked to sit on a chair with his arm resting on the table flexed at 90° to the axis of the trunk [42]. The examiner explained and demonstrated the protocol to the participant. Participants were asked to squeeze the dynamometer for a practice trial using submaximal effort to determine their understandings on the procedure and the grip size adjustments. The test was repeated 3 times on separately by 60 s and alternating hands. The final score was given by the mean of the 3 attempts.

Geriatric Depression Scale (GDS) [43,44] is a self-report scale used to assess the depressive symptoms. The scale consists of 30 items with a dichotomous response (“yes” or “no”), 20 items represent a depressed response if the answer is “yes” and 10 items represent a reverse score with a depressed response if the answer is “no”. The total score is given by the sum of the single item and ranged from 0 to 30 with a higher score indicating more depressive symptoms.

Basic Activities of Daily Living (BADL) was used to evaluate the autonomy in different daily living situations. In this study, the version of the test used was the 7 items scale proposed for the nursing home [45]. The activities evaluated were: dressing (how resident puts on, fastens and takes off all clothing), personal hygiene (how resident maintains personal hygiene), toilet use (how resident uses toilet room), locomotion on unit (how resident moves between locations in his/her room and the adjacent corridor on same floor), transfer (how resident moves between surface to/from bed, chair, standing position; exclude to/from toilet), bed mobility (how resident moves to and from lying position, turns side to side and places the body while in bed) and eating (how resident eats and drinks). Each individual activity was scored for 6 categories: “total independence” (score = 0), “supervision provided” (score = 1), “limited assistance” (score = 2), “extensive assistance” (score = 3), “total dependence” and “activity did not occur” (score = 4) (these two categories were combined into one as suggested in [46]). The total score is given by the sum of the single activity and ranged from 0 to 28, with a lower score indicating autonomy in daily living situations. The BADL were assessed by one researcher together with the doctor of the nursing.

### 2.5. Statistical Analyses

Means and standard deviations were calculated for all data, including the percent change for all outcome variables in both groups. All data were checked for normality using Shapiro–Wilk Test. T-test was used to check baseline differences between intervention and control group. Only for analytical purposes the intervention group was treated as having two arms (patients with and without dementia. The intervention group was divided in two: group 1 consisted of older adults with dementia and group 2 consisted of older adults without cognitive disease. Considering the sample size was greater than 30 (Glass et al., 1972; Lumley et al., 2002), a repeated measure analysis of covariance (ANCOVA) was performed for each dependent variable. Gender, age, BMI and baseline value were used as covariates for each variable. Differences between groups over time were determined by significant group X time interactions. Levene’s Test was used to assess homogeneity of variance (*p* > 0.050) and sphericity was assessed using Mauchly’s test (*p* > 0.050). If sphericity was violated the Greenhouse–Geisser correction was applied. The Statistical Package for Social Sciences (SPSS 24.0 for Windows) was used for all statistical analyses. The statistical significance level was set at *p* < 0.05.

## 3. Results

Table 1 presents the sociodemographic characteristics of both arms of the intervention group and the control group. The attendance to the activities were recorded by the nursing home staff and all the older adults in the intervention group participate to the 90% the activities and were able to reach the 150 min of physical activity every week. No missing data were found. However, as reported in Figure 1, few older adults were not able to complete the study, at different time points and as such, their data were not considered for the analysis. Shapiro–Wilk Test showed that all the variables were normally distributed (all *p* > 0.05). Back Scratch test showed a statistical difference between the intervention and control group at the baseline (R arm, *p* < 0.01; L arm, *p* < 0.05), in all the other variables there were no significant differences between the two groups at the baseline (all *p* > 0.05).

### 3.1. Mobility

**Figure 2:**
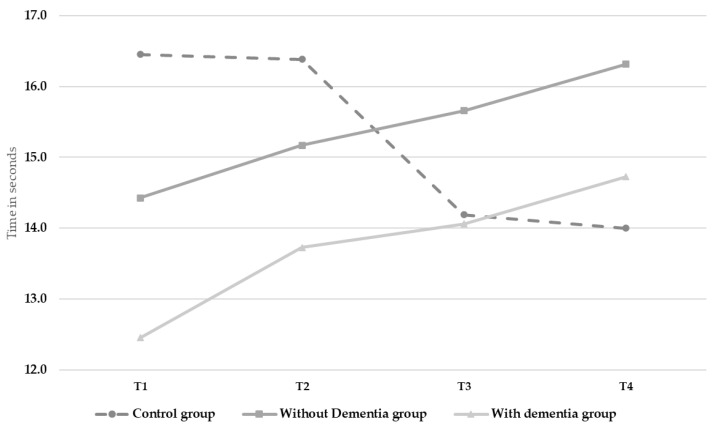
Changes in mobility (Tinetti test) over time.

There was a significant interaction effect between time and group for mobility (Tinetti test, F(187.265) = 17.775, *p* < 0.001, *η_p_*^2^ = 0.249). As shown in Figure 2, mobility decreased over time in the control group (from 16.45 to 14) but increased in both of the Intervention Group (I.G. without dementia from 14.43 to 16.31; I.G. with dementia from 12.45 to 14.73). There was also a significant interaction effect observed for the self-perceived fatigue ratings (F(4229.661) = 11.134, *p* < 0.001, *η_p_*^2^ = 0.171) which reflected an increase over time in the control group (from 3.95 to 5.36) whilst ratings for both intervention groups remained relatively stable across time (Table 2). The control variables showed no effects.

### 3.2. Strength

**Figure 3:**
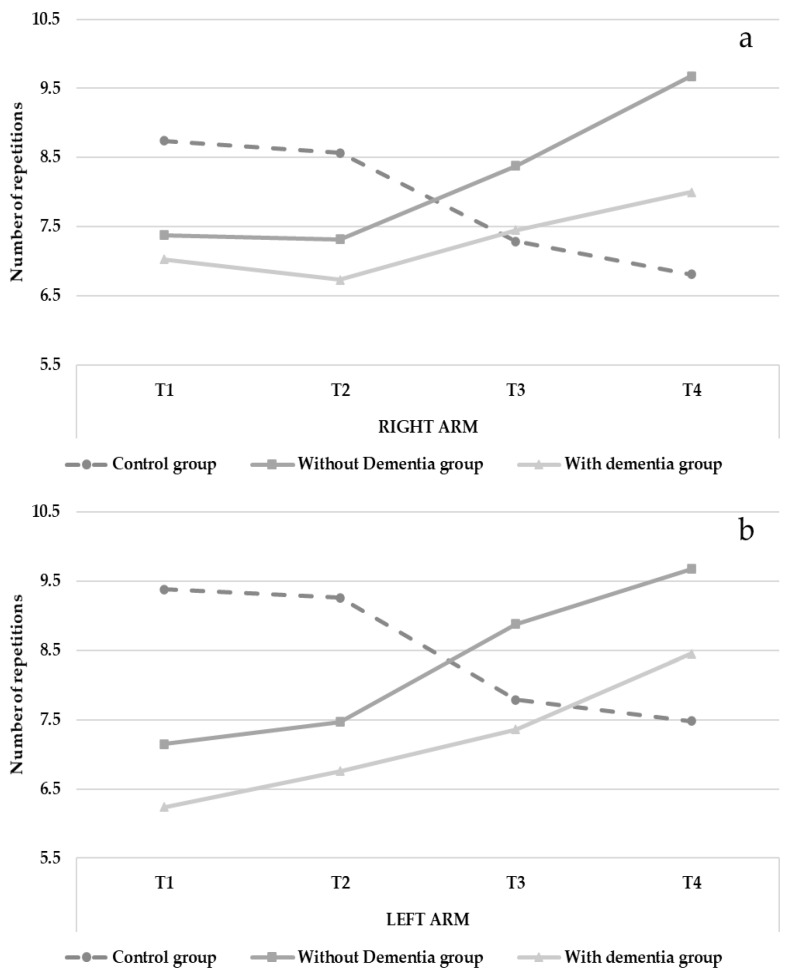
Changes in upper limb strength (Arm curl test) over time (right arm = **a**; left arm = **b**).

There were no significant interaction effects observed for the hand grip tests; however, the difference was statistically significant for the upper limb strength (Arm curl test: R arm, F(220.035) = 20.043, *p* < 0.001, *η_p_*^2^ = 0.274; L arm, F(181.640) = 31.45, *p* < 0.001, *η_p_*^2^ = 0.372). Upper limb strength decreased overtime in the control group (R arm from 8.74 to 6.81; L arm from 9.38 to 7.48) but improved over time in the intervention groups (I.G. without dementia: R arm from 7.38 to 9.68; L arm from 7.15 to 9.68; I.G. with dementia: R arm from 7.03 to 8.01; L arm from 6.24 to 8.45, See Figure 3 and Table 2). The control variables showed no effects.

### 3.3. Flexibility

**Figure 4:**
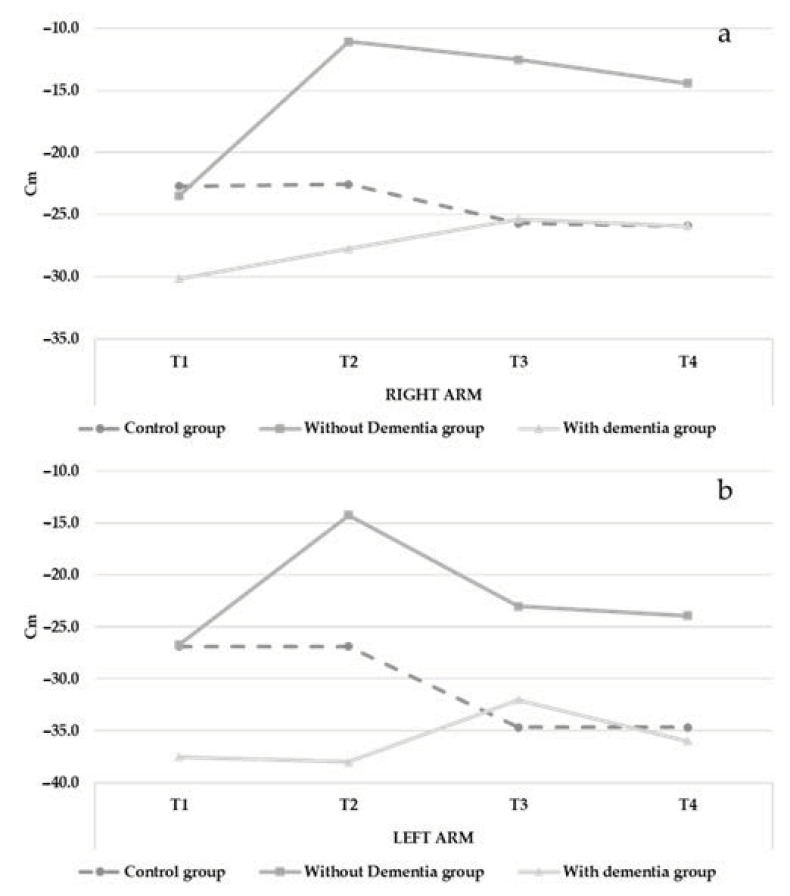
Changes in flexibility (Back Scratch test) over time (right arm = **a**; left arm = **b**).

Analysis of the back scratch test data revealed significant interaction effects observed in both arms (R arm, F(86.892) = 3.695, *p* < 0.05; L arm, F(220.035) = 4.013, *p* < 0.01). Flexibility and mobility of the shoulders decreased over time in the control group (R arm from −22.71 to −25.90; L arm from −26.86 to −34.67) but improved over time in the intervention groups (I.G. without dementia: R arm from −23.45 to −14.45; L arm from −26.70 to −23.90; I.G. with dementia: R arm from −30.12 to −27.87; L arm from −37.50 to −36.00, See Figure 4 and Table 2). The control variables showed no effects.

### 3.4. Autonomy and Depression

**Figure 5:**
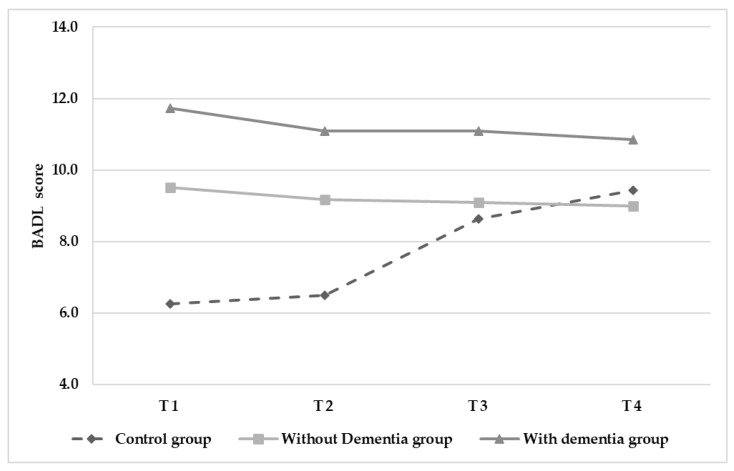
Changes in autonomy (BADL) score over time.

**Figure 6:**
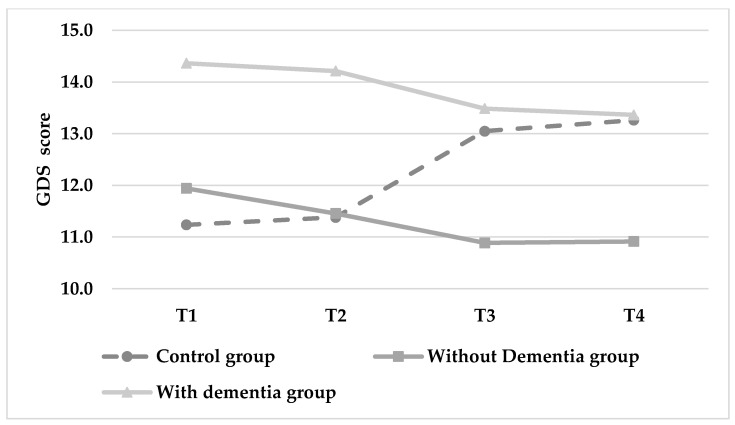
Changes in depression (GDS) score over time (lower score indicates greater autonomy in daily living situations).

Analyses revealed a significant interaction in BADL score (F(150.451) = 11.221, *p* < 0.001) resulting from observed decreases in BADL score for the control group (See Figure 5 and Table 2), whilst scores remained stable across time for both intervention groups. Finally, there was a significant interaction effect in depressive symptoms (F(144.222) = 15.640, *p* < 0.001). The control group showed an increased GDS score (from 11.24 to 13.26), while both intervention groups showed stable GDS scores across time (Figure 6 and Table 2). The control variables showed no effects.

## 4. Discussion

The general aim of this study was to investigate the effects of a 1 year lifestyle intervention on physical functioning, basic ADL and mood in older adults living in a long-term care setting. The main finding of our study was the preliminary efficacy of the intervention on the outcome variables and the acceptable adherence and safety of the training sessions. Our results clearly showed that participants in the intervention groups had significant improvements in overall mobility, upper limb strength and shoulder flexibility. They also remained stable in BADL score and depressive symptoms after 1 year of intervention. These results should be cautiously interpreted because of the small sample size of our study. However, these are important results considering the institutionalized condition of the participants living in the nursing home and the positive effect of the intervention on the older adults with and without dementia.

The findings of the present study support previous research that has pointed to the positive effects that exercise based interventions are an effective strategy in maintaining functional performance of older adults and are vital for maintaining functional independence [47,48]. The results also demonstrated significant increases in some of the strength parameters that were measured, namely arm curl strength, following the introduction of the intervention. In comparison, the control group observed significant decreases across the same time frame. This decrease is in line with previous research that highlights how reductions in skeletal muscle mass is also accompanied by a significant decrease in muscle strength during ageing [49,50]. These findings have significant applied implication given the importance muscle strength plays as a determinant of healthy ageing [51,52]. Indeed, the loss of muscle strength has been shown as a principal indicator for many geriatric syndromes, including weakness syndrome, sarcopenia, mobility impairments and falls [53]. However, it should be noted that measures used in this research revealed no significant findings in the present investigation (hand-grip strength).

The intervention also proved successful in reducing the observed decline in older adults’ flexibility. Research has suggested that shoulder abduction declines by five degrees/decade in men and six degrees/decade in women on average [54]. Upper body flexibility, measured in the present investigation, is essential in executing vital day to day activities such as getting dressed or reaching for objects [55,56] and directly impacts older adults dependence upon staff in long-term healthcare settings.

The results of this study highlight the importance of physical activity within care home settings through improving aspects of both physical and mental wellbeing of the residents. Engaging with physical activity requires both a willingness to move and the ability to perform a task, whether it is simply standing up from a chair or undertaking more vigorous activity. In care home settings, both of these may be impacted through limited autonomy of the residents and limited capacity of the staff to assist them. Indeed, previous research has highlighted that the percentage of residents who participate in regular exercise was lower than the reported percentage of older adults who exercise regularly in the community [57]. However, through engaging in a physical activity intervention, we have shown that residents physical abilities in terms of general mobility and upper body strength will improve. This may allow them to be more autonomous and less reliant on staff to perform basic movements and daily activities.

This is supported through the fact that BADL performance among the intervention groups remained stable whereas it reduced in the control group. It has been shown that the ability to perform BADL is the most important factor when assessing the quality of life of those with dementia [58]. An individual’s quality of life is closely linked with their motivation to engage with physical activity [25,58] and thereby their ability to remain mobile and continue performing BADL autonomously [59,60]. Through enabling physical activity in care homes, this cycle can be maintained, leading to positive mental and physical benefits for residents.

It is known that a sedentary lifestyle can lead to negative mental health effects, especially related to depressive symptoms and, therefore, it can be suggested that depressive symptoms may reduce by increasing physical activities [61]. Research has previously demonstrated an independent relationship between depression status and longitudinal change in functional capacity among community-dwelling older individuals [62]. Although this study showed that depressive symptoms remained stable among the intervention group, they increased in the control group and, therefore, the intervention can still be interpreted as having a positive outcome. However, the course of depression in older adults living in care homes are still unclear and current evidence is mixed [63]. Indeed, some studies suggest that symptoms tend to persist [64] or deteriorate [65]; in contrast, Payne et al. [66] found that depression decreased after 1 year for the admission in a care home. The same is true for self-perceived fatigue, which also increased among the control group but remained stable in the intervention groups. Feeling fatigued leads to a preference for easier tasks such as sitting rather than expending energy [67], which in turn increases sedentary behaviour and, subsequently increased self-perceived fatigue. Although the intervention in this study did not reduce the feelings of fatigue, it also did not lead to an increase, which again allows this to be interpreted as a positive result.

When comparing the intervention group to the control group, the variation between pre-test and post-test data in relation to the age of the sample and to the institutionalized condition, highlights the protective function played by physical activity on the health of the older elderly, for whom a good overall physical condition is a key precondition for an independent daily life. Facing and accepting the challenges of a changing body, rediscovering the joys of movement and socializing, as well as once again learning to trust oneself, preserving one’s skills and applying them to daily living in order to retain one’s autonomy as long as possible all of this can be turned into a great chance to promote health and to prevent a wide range of risks faced by older adults.

Several potential limitations of this pilot study must be considered. The participants were not randomly allocated to the intervention and control groups because of management and logistic problems of the residential care facilities. The relatively small sample size did not allow us to generalize our results to a larger older population living in long-term care settings; further studies using larger sample sizes are needed to expand this study and make solid inferences of the benefits of the intervention. The instructor together with the NH1 staff monitored the adherence to the activity and did not report any particular problem. However, there are no data that can confirm the implementation fidelity for each participant and session during the whole year of intervention. In order to obtain these data, a system capable of automatically measuring and analysing the activities should be used, such as the WBR-SH2 [68] or similar. An additional limitation is related to the fact that no follow-up was carried out to evaluate maintenance of an active behaviour positive effects over time; the follow up was planned after 1 year from the end of the intervention, but this was not possible due to the restrictions related to COVID19 pandemic.

## 5. Conclusions

Our findings demonstrated that 1 year lifestyle intervention based on the WHO physical activity recommendations for older adults (minimum 150 min of physical activity per week) was effective for individuals living in a long-term care setting; indeed, the implementation of this program may improve physical functioning, basic ADL and mood in older adults with and without dementia. Therefore, the engagement and inclusion of a lifestyle intervention in the schedule of these care facilities and in the daily routine of older residents in long-term care settings may improve the quality of life.

## Figures and Tables

**Table 1:** Sample demographic characteristics by group.

	Experimental Groups		Control Group n = 42 (37.8%)
	Without Dementia n = 35 (31.5%)	With Dementia n = 34 (30.6%)	
Gender, n (%)			
Male	7 (20.0%)	2 (5.9%)	5 (11.9%)
Female	28 (80.0%)	32 (94.1%)	37 (88.1%)
Age, mean ± SD	80.57 ± 9.65	84.88 ± 7.36	81.67 ± 10.55
Weight, mean ± SD	65.76 ± 13.73	57.64 ± 12.36	58.35 ± 10.34
Height, mean ± SD	154.75 ± 19.34	155.11 ± 8.00	154.44 ± 7.37
Body Mass Index (BMI), n (%)			
Underweight	1 (2.9%)	6 (17.6%)	0 (0%)
Normal weight	15 (42.9%)	12 (35.3%)	26 (61.9%)
Pre-obesity	9 (25.7%)	10 (29.4%)	12 (28.6%)
Obesity class 1	10 (28.6%)	6 (17.6%)	4 (9.5%)
Ethnicity, n (%)			
Italian	35 (100%)	34 (100%)	42 (100%)
Marital Status, n (%)			
Single	5 (14.3%)	7 (20.6%)	9 (21.4%)
Married	4 (11.4%)	3 (8.8%)	1 (2.4%)
Widowed	26 (74.3%)	24 (70.6%)	32 (76.2%)
Education, n (%)			
Primary school	18 (51.4%)	23 (67.6%)	24 (57.1%)
Secondary school	13 (37.1%)	9 (26.5%)	14 (33.3%)
High school	4 (11.4%)	2 (5.9%)	4 (9.5%)
Previous Work, n (%)			
Manual	28 (80.0%)	26 (76.5%)	36 (85.7%)
Non manual	7 (20.0%)	8 (23.5%)	6 (14.3%)

**Table 2:** Analysis of variance testing the effect of 1 year lifestyle intervention on all the outcome variables.

Test	Group	Pre-Test	1° Test	2° Test	Post-Test	Percentual Variation Pre-Test Post-Test	F	Partial Eta Square	Sig. *p* < 0.05
Tinetti Test	I.G. without dementia	14.43 ± 9.51	15.17 ± 9.49	15.66 ± 9.82	16.31 ± 9.79	+13.03%	17.775	0.249	0.001
	I.G with dementia	12.45 ± 9.54	13.73 ± 9.62	14.06 ± 9.57	14.73 ± 9.67	+18.31%			
	C.G.	16.45 ± 8.93	16.38 ± 8.91	14.19 ± 9.30	14.00 ± 9.28	−14.89%			
Borg Scale	I.G. without dementia	4.09 ± 2.32	4.63 ± 2.50	4.40 ± 2.37	4.17 ± 2.09	+1.96%	11.134	0.171	0.001
	I.G. with dementia	5.32 ± 2.16	5.21 ± 2.32	5.00 ± 2.17	4.88 ± 2.11	−8.27% *			
	C.G.	3.95 ± 2.48	3.93 ± 2.42	5.33 ± 1.96	5.36 ± 2.03	+35.70%			
Arm Curl Test Right (R)	I.G. without dementia	7.38 ± 2.71	7.32 ± 2.79	8.38 ± 2.93	9.68 ± 2.84	+31.17%	20.043	0.274	0.001
	I.G. with dementia	7.03 ± 3.03	6.73 ± 3.06	7.45 ± 3.13	8 ± 3.40	+13.94%			
	C.G.	8.74 ± 2.79	8.57 ± 2.66	7.29 ± 2.30	6.81 ± 2.19	−22.08%			
Arm Curl Test Left (L)	I.G. without dementia	7.15 ± 2.81	7,47 ± 2.65	8.88 ± 3.07	9.68 ± 3.01	+35.38%	31.45	0.372	0.001
	I.G with dementia	6.24 ± 3.28	6,76 ± 3.22	7.36 ± 2.89	8.45 ± 2.85	+35.41%			
	C.G.	9.38 ± 3.02	9.26 ± 2.92	7.79 ± 3.04	7.48 ± 2.95	−20.26%			
Hand Grip Test Right (R)	I.G. without dementia	16.54 ± 5.33	16.54 ± 4.18	16.54 ± 4.18	16.85 ± 4.74	+1.87%	0.317	0.012	0.868
	I.G. with dementia	12.47 ± 4.88	11.30 ± 4.00	11.22 ± 3.91	11.33 ± 3.98	−9.14%			
	C.G.	15.24 ± 5.46	14.64 ± 5.12	14.44 ± 4.92	14.97 ± 5.29	−1.77%			
Hand Grip Test Left (L)	I.G. without dementia	14.31 ± 4.33	15.38 ± 4.17	14.38 ± 4.43	15.77 ± 3.98	+10.20%	0.404	0.015	0.747
	I.G. with dementia	10.77 ± 3.27	11.54 ± 2.85	10.92 ± 3.23	11.54 ± 2.99	+7.15%			
	C.G.	13.71 ± 4.25	14.71 ± 3.49	13.84 ± 4.28	13.94 ± 3.47	+1.68%			
Back Scratch Test Right (R)	I.G. without dementia	−23.45 ± 15.08	−11.09 ± 14.70	−12.54 ± 14.27	−14.45 ± 14.10	−38.38% *	3.695	0.113	0.013
	I.G. with dementia	−30.12 ± 13.90	−27.75 ± 10.99	−25.37 ± 8.94	−27.87 ± 11.77	−7.47% *			
	C.G.	−22.71 ± 14.65	−22.56 ± 14.52	−25.69 ± 14.57	−25.90 ± 13.06	+14.05%			
Back Scratch Test Left (L)	I.G. without dementia	−26.70 ± 16.26	−14.20 ± 17.99	−23.00 ± 13.00	−23.90 ± 14.87	−10.49% *	3.858	0.123	0.011
	I.G. with dementia	−37.50 ± 12.91	−38.00 ± 7.54	−32.00 ± 9.21	−36.00 ± 11.90	−4.00% *			
	C.G.	−26.86 ± 15.95	−26.86 ± 15.95	−34.67 ± 13.98	−34.67 ± 13.98	+29.07%			
BADL	I.G. without dementia	9.51 ± 8.88	9.17 ± 8.85	9.09 ± 8.83	9.00 ± 8.82	−5.36% *	11.221	0.172	0.001
	I.G. with dementia	11.74 ± 7.41	11.09 ± 7.61	11.09 ± 7.68	10.85 ± 7.77	−7.58% *			
	C.G.	6.26 ± 5.21	6.50 ± 5.24	8.64 ± 5.79	9.43 ± 6.00	+50.64%			
GDS	I.G. without dementia	11.94 ± 5.56	11.46 ± 6.06	10.89 ± 6.10	10.91 ± 6.21	−8.63% *	15.640	0.226	0.001
	I.G. with dementia	14.36 ± 5.33	14.21 ± 5.27	13.48 ± 5.29	13.36 ± 5.45	−6.96% *			
	C.G.	11.24 ± 5.22	11.38 ± 5.05	13.05 ± 5.19	13.26 ± 5.21	+17.97%			

Note: I.G. = Intervention group, C.G. = Control group. * Negative percentage indicate an improvement in the task.

## Data Availability

Data are available upon request.
